# Sexually selected sentinels? Evidence of a role for intrasexual competition in sentinel behavior

**DOI:** 10.1093/beheco/arw064

**Published:** 2016-04-24

**Authors:** Lindsay A. Walker, Jenny E. York, Andrew J. Young

**Affiliations:** ^a^Centre for Ecology and Conservation, University of Exeter, Cornwall Campus, Treliever Road, Penryn, Cornwall TR10 9EZ, UK and; ^b^Department of Zoology, University of Cambridge, Downing Street, Cambridge CB2 3EJ, UK

**Keywords:** intrasexual competition, *Plocepasser mahali*, sentinels, sexual selection, vigilance.

## Abstract

Although the evolutionary mechanisms that favor investment in cooperative behaviors have long been a focus of research, comparatively few studies have considered the role that sexual selection may play. For example, evolutionary explanations for sentinel behavior (where 1 individual assumes an elevated position and scans the surroundings while other group members forage nearby) have traditionally focused on the inclusive fitness benefits arising from its effects on predation risk, while its potential role in defense against intrasexual competitors remains largely unexplored. Here, we provide experimental evidence of a role for sentinel behavior in intrasexual competition, in a cooperatively breeding songbird, the white-browed sparrow weaver (*Plocepasser mahali*). First, dominant males sentinel substantially more than other group members (even when controlling for variation in age and body condition), consistent with a role for sentineling in intrasexual competition for mates and/or territory. Second, experimental playback of an unfamiliar male’s solo song elicited a marked increase in sentineling by the dominant male, and the vocal response to the playback also positively predicted his sentinel effort following the simulated intrusion. A second experiment also suggests that sentineling may facilitate mounting rapid anti-intruder responses, as responses to intruder-playback occurred significantly earlier when the dominant male was sentineling rather than foraging at playback onset. Together, our findings provide rare support for the hypothesis that sentinel behavior plays a role in intrasexual competition, and so highlight the potential for sexually selected direct benefits to shape its expression in this and other social vertebrates.

## Lay Summary

Few studies have considered whether competition between members of the same sex has shaped the expression of cooperative behaviors. Here, we provide experimental evidence that sentinel behavior plays a role in defense against same-sex competitors. Song playbacks that simulated male intrusions elicited a marked increase in sentineling by the dominant male, and also suggested that the sentinel position itself may facilitate the initiation of anti-intruder responses.

Twitter: @Linds__Walker @animalsocieties

## INTRODUCTION

Although the evolutionary mechanisms that favor investment in cooperative behaviors have long been a focus of research, comparatively few studies have investigated the role that sexual selection may play (Reyer 1990; Dickinson and Hatchwell 2004; Boomsma 2007; DuVal 2013). For example, contributions to sentinel behavior (where 1 individual watches from an elevated position while other group members forage nearby) are widely considered to reflect inclusive fitness benefits arising from sentineling serving an antipredator function. Indeed, a wide range of evidence supports this view: Sentinels frequently alarm call at predators (Ferguson 1987; Beynon and Rasa 1989; McGowan and Woolfenden 1989; Clutton-Brock 1999; Wright et al. 2001a), sentinels may detect predators more readily than foraging group members (Rasa 1986; Manser 1999; Ridley et al. 2010), and individuals may sentinel at higher rates in response to increased predation risk (Ferguson 1987; Clutton-Brock 1999; Ridley et al. 2010; Radford et al. 2011; Santema and Clutton-Brock 2013; Kern and Radford 2014). However, individual contributions to sentineling could also be attributable in part to fitness benefits accrued through alternative mechanisms, such as a role for sentineling in intrasexual competition. For example, sentineling could provide a vantage point for detecting and responding to same-sex competitors, who might otherwise threaten a resident individual’s reproductive success and/or social dominance. Where this is the case, sexually selected direct benefits may play a key role in shaping contributions to sentinel behavior.

Consistent with a role for sentineling in intrasexual competition over matings, studies of individual contributions to sentinel behavior in several social species have found that males sentinel at higher rates than females (e.g., Florida scrub jay, *Aphelocoma coerulescens coerulescens*: Hailman et al. 1994; vervet monkey, *Cercopithecus aethiops*: Baldellou and Peter Henzi 1992; Arabian babbler, *Turdoides squamiceps*: Wright, Maklakovi, et al. 2001; meerkat, *Suricata suricatta*: Clutton-Brock et al. 2002). These findings highlight the possibility that sentinel behavior yields differential benefits to males through a mechanism other than the mitigation of predation risk. Sentineling could enhance a male’s ability to detect, repel, and advertise his presence to same-sex competitors who might otherwise contest his paternity and/or social dominance. Although females too may certainly benefit from vigilance against same-sex intruders (as intrasexual competition for dominance status in cooperatively breeding vertebrates may be at least as intense among females as males; Clutton-Brock et al. 2006; Young and Bennett 2013), males may frequently enjoy greater benefits from doing so given the threat that even transient males may pose to a resident male’s paternity (e.g., Young et al. 2007; Harrison et al. 2013b). As most of the species that sentinel typically live in extended family groups (e.g., dwarf mongoose, *Helogale parvula*: Rasa 1986; Florida scrub jay: McGowan and Woolfenden 1989; Arabian babbler: Wright et al. 2001a, Wright, Maklakovi, et al. 2001; white-browed sparrow weaver, *Plocepasser mahali*: Harrison et al. 2013b), threats to a male’s reproductive monopoly may principally be posed by unrelated extra-group males, whether prospecting males (who may threaten both paternity and dominance; Young et al. 2007; Mares et al. 2011) or extra-group resident dominants (who may constitute the principal threat to another dominant’s paternity; Richardson et al. 2001; Harrison et al. 2013b). Indeed, observations that dominant male Arabian babblers sentinel at the highest rates, and emit territorial calls at higher rates than other classes when sentineling, led Wright, Maklakovi, et al. (2001) to suggest that such males may accrue additional benefits from sentineling if it facilitates monitoring and calling to neighboring groups. Sentineling may also facilitate the monitoring and mitigation of within-group threats to a male’s reproductive monopoly, which may be more acute in instances where more complex kin structures leave resident subordinate males unrelated to 1 or more resident females (Koenig and Haydock 2004; Young 2009). It has also been suggested that fitness costs entailed in sentineling could leave an individual’s sentinel effort an honest signal of their quality (Zahavi A and Zahavi A 1997, see also Wright 1999). Any role for sentineling in intrasexual competition could therefore conceivably extend beyond facilitating the detection and repulsion of same-sex competitors, to signaling the sentinel’s quality to those competitors and/or the mates for which they compete. Whether sentineling does play a role in intrasexual competition, however, has yet to be formally tested.

Here, we investigate whether sentinel behavior plays a role in intrasexual competition, using a combination of observational data sets and playback experiments in a wild population of the white-browed sparrow weaver, a year-round territorial cooperatively breeding songbird. White-browed sparrow weavers live in groups of 2–12 individuals, consisting of a dominant breeding pair and subordinates of both sexes (Collias NE and Collias EC 1978; Lewis 1982; Ferguson 1988; Harrison et al. 2013a). Sentinel behavior in white-browed sparrow weavers, as for other ground-foraging species, is characterized by an individual assuming a raised position and scanning its surroundings while members of its group forage nearby (Ferguson 1987). Previous work has attributed an antipredator function to sentinel behavior in this species, on the basis of sentinels emitting alarm calls when predators are detected and alarm calling at higher rates than nonvigilant birds, and individuals showing higher rates of sentinel behavior in higher risk microhabitats (Ferguson 1987). Ferguson (1987) also reported that sentineling individuals produced territorial vocalizations at significantly higher rates than perched nonvigilant birds, highlighting the possibility of an additional role for sentineling in competition with extra-group individuals over territory, group membership, and/or reproductive opportunities (see also Wright et al. 2001a). Extra-group males pose the principal threats to a dominant male white-browed sparrow weaver’s reproductive monopoly and social dominance (Harrison et al. 2013a, 2013b). Within-group subordinate males, including immigrant individuals, have never been known to secure paternity within their social group (Harrison et al. 2013a); dominant males do lose 12–18% of paternity, but do so exclusively to extra-group males (principally dominant males in other groups; Harrison et al. 2013b). Likewise, dominant males appear rarely to be usurped by natal subordinate males, with immigrant or extra-group males assuming dominance in 82.7% of monitored dominance turnovers (Harrison et al. 2014). Although dominant females also principally lose dominance to extra-group females (88% of monitored dominance turnovers; Harrison et al. 2014), extra-group females may pose little immediate threat to a dominant female’s maternity, as egg-dumping has never been detected in this species; the dominant female invariably monopolizes reproduction (Harrison et al. 2013a). Consequently, dominant male white-browed sparrow weavers might be predicted to benefit more from investing in the detection and repulsion of same-sex intruders than dominant females. Whether sentinel behavior does play a role in intrasexual competition, perhaps by facilitating the detection and/or repulsion of such extra-group threats, is not known.

Specifically, we test the hypothesis that sentinel behavior plays a role in intrasexual competition, by addressing the following 3 specific aims. First, we use observational data to investigate whether dominant male white-browed sparrow weavers contribute more to sentinel behavior than other group members (while controlling for variation in age and body mass), as would be predicted if it conveys advantages in competition against extraterritorial threats to the dominant’s reproductive monopoly and/or social dominance. Second, we experimentally investigate whether dominant males increase their investment in sentinel behavior in response to the playback of an unfamiliar male’s solo song relative to paired control playbacks. White-browed sparrow weaver males sing a distinct song type during the breeding season, known as the solo song (see Voigt et al. 2006 for a spectogram of solo song). It is sung principally by dominant males, typically at dawn but also during the day, particularly during peak reproductive periods following high rainfall (Wingfield and Lewis 1993; Voigt et al. 2006; York 2012; York et al. 2016). Extra-group males are known to intrude on occupied territories, where they have been observed producing solo song, eliciting vocal and physical responses (chases) from the resident dominant male. The playback of a foreign male’s solo song is therefore likely to be indicative of the intrasexual competitive threat posed by extra-group males. Finally, we investigate whether sentineling may also facilitate such counter-intruder responses, by establishing whether dominant males mount faster responses to foreign male solo song playbacks when individuals are acting as sentinels than when foraging.

## METHODS

### Study species and population

Data were collected from a color-ringed study population at Tswalu Kalahari Reserve in the Northern Cape province of South Africa (27°16′S, 22°25′E; see Harrison et al. 2013a for a detailed site description) between December 2012 and March 2014. All birds were fitted with a metal ring and 3 color rings for identification under SAFRING license 1444. Males and females are readily distinguished in the field from around 6 months of age by beak color; males have dark-brown beaks with females displaying a paler horn color (Leitner et al. 2009; Harrison et al. 2014). All individuals in the study were semi-habituated to observation with telescopes at approximately 18–20 m, following 5 years of regular exposure to observers at this distance. The dominant bird of each sex was determined by weekly monitoring of dominance-related aggressive, displacement, and reproductive behaviors (as outlined in Harrison et al. 2013a; York et al. 2014). All protocols were approved by the University of Pretoria Ethics Committee and complied with regulations stipulated in the Guidelines for Use of Animals in Research.

### Natural sentinel behavior observations

Throughout the study, individuals were defined as engaging in sentinel behavior (following Ridley et al. 2010), if they were perched in an elevated position >1 m above the foraging group members, actively scanning the surrounding area for a duration of >30s. Similarly to pied babblers (*Turdoides bicolor*, Ridley et al. 2010), white-browed sparrow weavers are predominately ground foragers (Ferguson 1988), with individuals using their beaks to dig in the substrate for prey. As sentinel behavior was conspicuous and occurred at low frequencies (see Results for details, white-browed sparrow weaver groups had a sentinel in place a mean [± standard deviation {SD}] of 21.0% [± 7.80] of the time, and individuals did not overlap in sentinel bouts), the sentinel effort of all group members could be accurately monitored simultaneously simply by noting the start and end times of all sentinel bouts during the observation session. All sentinel observation sessions were performed between 06:45 and 10:00, during the breeding season (October–April), and at times when groups lacked nestlings (so as to minimize the impact of trade-offs between concurrent reproductive investment and sentineling on patterns of sentinel effort). On first locating each focal group, a period of at least 15min was used to establish group composition and to allow the birds to habituate to the observer, prior to beginning the sentinel observation session.

In order to contrast the sentinel effort of the different dominance and sex classes (to address the first aim), 53 sentinel monitoring observation sessions were conducted on 25 social groups (2–6 individuals per group, mean 3.85 individuals, with social groups visited on 1–4 separate observation sessions) that comprised a total of 25 dominant males and females, 28 subordinate males, and 11 subordinate females. Following the 15-min habituation period, sentinel observation sessions were conducted for 30 or 60min (according to logistical constraints) and the length of the observation session (short or long) was fitted as a random effect in our statistical models to control for any effect it might have on the proportion of time spent sentineling. To then investigate whether the higher sentinel effort of dominant males relative to subordinate males (as revealed by analysis of the above data set) could be driven by associated variation in body condition, the sentinel effort of dominant and subordinate males was also quantified with a separate sample of 60-min observation sessions (*n* = 12; from 12 social groups, containing a total of 11 dominant males and 10 subordinate males) all collected within a 3-day window (mean [± SD] = 0.94±1.25 days) prior to the collection of matched morphometric data for these birds (from which body condition could be calculated; see below). This second data set was collected exclusively during periods when groups were incubating (days 4–13 of incubation; invariably after clutch completion), as this is when all of our study groups are routinely captured for the collection of morphometric data (males do not incubate in this species; Harrison et al. 2013a).

### Body condition calculation

Birds were captured at night by flushing them from their individual roost chambers into a custom capture bag and were subsequently returned to their roost chambers the same night by hand (Cram et al. 2015). Body mass was recorded to the nearest 0.01g (Durascale 100; MyWeigh, Phoenix, AZ) and tarsus length measured ±0.1mm using calipers; all measurements were taken by 1 person (L.A.W.). To investigate the effect of body condition on individual sentinel effort, we calculated the scaled mass index (SMI) as a proxy for body condition (following Peig and Green 2009), as SMI has been argued to perform better than other methods of estimating body condition (Peig and Green 2009). Calculations of SMI entail the estimation of a scaling exponent (standardized major axis [SMA]) from a reduced major axis regression of the logarithm of body mass on the logarithm of tarsus length (in this study, SMA = 0.22). The body mass values of all birds were then scaled to the expected equivalent for a bird of the mean tarsus length in our sample (24.88mm), using the SMA (for full details, see Peig and Green 2009). This SMI value for each bird was then used in our analysis as a proxy for body condition.

### Playback experiments

We conducted 2 playback experiments with similar designs. For both experiments, a paired within-individual design was utilized to control for interindividual differences, with each focal dominant male being exposed to one of 2 treatments on 1 day and the other treatment on the subsequent day, with treatment order reversed for each successive individual. All playback trials were initiated via a wireless connection with a media player (Philips Android Connect) once specific conditions were satisfied (see details below). Playbacks were conducted using Jawbone (Jambox) portable speakers placed at a height of 1.5 m on the main sleeping roost tree at the center of the focal group’s territory at an amplitude of 66 dB (Voltcraft SL100 digital sound level meter, Voltcraft, Barking, UK) at 10 m from the speaker (to simulate the average amplitude of natural song as measured at this distance). The observer (L.A.W.) was stationed at 20 m from the playback speaker throughout the session. All behavioral observations (details provided below) were dictated and recorded on a DM550 Olympus recorder (ME15 Olympus microphone), and they were subsequently examined using Raven Lite 1.0 (Cornell Laboratory of Ornithology, Ithaca, NY, 2006).

The playback audio tracks were produced using CoolEditPro 2.0 (Syntrillium Software Corporation) from solo song recordings collected from 10 different dominant males (York 2012; York et al. 2014) using a Sennheiser ME66 directional microphone with a K6 power module (2004 Sennheiser) and a Marantz PMD660 solid-state recorder (DandM Holdings Inc.) and used for both experiments. Each playback track consisted of either a 3-min continuous section of male solo song (male solo song treatment) or a 3-min continuous section of the ambient background sounds recorded on the same track (control treatment), resulting in 10 pairs of unique male solo song tracks and control tracks each from a different original male. Three minutes were selected as the standard duration for the playback stimulus for 2 reasons. First, a 3-min duration of male solo song is within the bounds of natural song produced outside of the dawn period (male solo song produced outside of the dawn period has been observed to range from 20s up to 6min, but occurs less frequently than male solo song production at dawn). Second, the intention was to provide a moderate stimulus that would not excessively stimulate the focal male to the point of habituation and/or triggering artificial responses. Given that the playback duration was standardized for all stimuli and for both treatments, a 3-min stimulus duration provided an ecologically relevant and standardized stimulus duration for these experiments. Care was taken to ensure that the playback tracks included either only male white-browed sparrow weaver solo song (male solo song treatment) or no conspecific vocalizations at all (control tracks) to avoid receiver responses to other conspecific vocalizations. So as to ensure that the source-male for each playback track was unfamiliar to the resident male to whom the track was played, we ensured that the recording and playback territories for each track were at least 3 territories apart (mean ± SD: 859±241 m).

#### Experiment 1: does an intrasexual challenge impact dominant male sentinel behavior?

To investigate whether sentinel behavior may function in intrasexual competition, we simulated the presence of an unseen same-sex (male) intruder by conducting a 3-min audio playback of an unfamiliar male’s solo song (a song type performed principally by dominant males during the breeding season, but that can also be sung by subordinate males and intruders; York 2012) and contrasted the behavioral response elicited with that elicited by a control playback. We conducted the playbacks on 10 white-browed sparrow weaver pairs (i.e., territorial groups containing just 1 dominant male and 1 dominant female with an established pair bond) to eliminate any potential complications arising from the presence of resident subordinates (e.g., competitive or compensatory responses to the sentinel effort of subordinates). All of the playbacks were conducted during the incubation phase of breeding, but while both the male and female were foraging together away from their nest. As dominant females do not display sentinel behavior during incubation periods (Walker LA, Young AJ, unpublished data), this approach ensured that any changes in the dominant male’s sentinel behavior following playback could not be attributed instead to him modifying his sentinel effort in response to an effect of the playback on the sentinel effort of the dominant female. That said, this approach could conceivably elicit larger responses from the dominant male than might be anticipated if the experiment was conducted on groups containing subordinates, where other individuals might also be expected to respond.

The experiment proceeded as follows. Each focal pair received 1 treatment per day over 2 successive days, with the order of presentation of the solo song and control treatments alternated for each successive pair. Two focal pairs were visited on each experimental day and they received the opposite treatments. All observation sessions began between 05:45 and 08:30. Following the completion of an initial 15-min habituation period, all sentinel activity (start and end times for all sentinel bouts) by the resident pair was recorded for 30min. The playback was then started once the foraging pair moved in front of the portable speaker. The distance of the resident pair to the speaker when playback was initiated was estimated by first noting their location on a detailed territory map and then taking a GPS location of that point once the observation session was complete. There was no significant difference in the distance from the speaker to the foraging pair between the 2 playback treatments (mean [± SD] for song treatment: 51.67 [± 28.40] m and control treatment: 43.11 [± 19.90] m; generalized linear model [GLM]: $\chi_{1}^{2}$ = 0.66, *P* = 0.41). While the track played (for 3min), the following behavioral responses and the identity of the individuals involved were recorded: 1) song production (all incidences of male solo song and duets [a distinct song repertoire mainly produced by the dominant pair; Voigt et al. 2006]) and 2) territory movements: leading movements (defined as 1 group member leaving and being promptly followed by the other, traveling in the same direction and arriving at same destination) and approach to playback tree (defined as landing in the tree containing the speaker). Once the 3-min playback was complete, a second 30-min post-playback behavioral observation session was conducted, in which the sentinel efforts of both group members were recorded (as per the session prior to the playback).

#### Experiment 2: does sentineling facilitate responses to an intrasexual challenge?

To determine whether the position of the dominant male (sentineling vs. foraging on the ground) affects his latency to respond to a perceived territorial threat, we simulated the presence of a same-sex intruder in the 2 contexts by conducting a 3-min playback of unfamiliar male solo song (according to the playback methods described above). This experiment was conducted during the breeding season, but during periods when the focal group did not have eggs or nestlings present. As for experiment 1, the playbacks were conducted on the territories of resident pairs (*n* = 8). Each pair received a different male solo song playback track, and the same track was utilized in both contexts for the same pair (so as to standardize across contexts any impact the specific song elements might have on the scale of the perceived threat).

All 8 resident pairs were visited on 1 day for their first treatment and again the next day for the opposite treatment, with the pairs successively allocated alternate first treatments (male sentineling vs. male foraging). All observation sessions occurred between 06:45 and 12:05. In each case, following a 15-min habituation period, the 3-min male solo song playback was begun once the dominant male was either 1) participating in sentinel behavior for ≥30s or 2) foraging with his social mate for ≥30s. The distance of the resident pair to the speaker when playback was initiated was estimated by first noting their location on a detailed territory map and then taking a GPS location of that point once the observation session was complete. There was no difference in the distance from the speaker to the dominant male between the 2 playback contexts (mean [± SD] for sentinel condition: 36.73 [± 11.40] m and foraging condition: 35.68 [± 11.10] m; GLM: $\chi_{1}^{2}$ = 0.029, *P* = 0.87). During the 3-min playback and the ensuing 10min, behavioral responses and the identity of the individuals involved were recorded exactly as outlined for experiment 1 above. Four of the resident pairs used in this experiment were also used in experiment 1, but there were at least 17 days (mean [± SD] = 49 [± 27.12] days) between their exposures to male solo song playback, and the male solo song tracks used for these groups were different in the 2 experiments.

### Statistical analyses

All statistical analyses were conducted with R v 3.0.3 (R Development Core Team 2014). All models were checked for normality of residuals, homogeneity of variance, and overdispersion. Statistical modeling utilized a stepwise model simplification approach: Initially, all fixed terms (detailed below) were fitted together and then the nonsignificant terms with the least explanatory power were sequentially removed until a minimal adequate model was reached (retaining only those predictors whose removal now yielded a significant reduction in the explanatory power of the model). All random terms (detailed below) were retained in the model throughout. The assessments of statistical significance reported for each of the fixed terms are those calculated from the change in explanatory power on removal of that term from the minimal model (if it was in the minimal model) or following its inclusion in and subsequent removal from the minimal model (if it had not been retained in the minimal model). See Table 1 for full details of all explanatory variables investigated in each model.

**Table 1:** Model outputs of mixed-effect models as detailed in the appropriate analyses

Model terms	Estimate	SE	df	*P*	Sample size	Constant
Natural sentinel data: do dominant males invest differentially in sentinel behavior?						−1.64
Sex × dominance	Figure 1		1	0.031	200	
Social group size	0.0095	0.064	1	0.86	200	
Natural sentinel data: is the dominance effect among males attributable to age?						−0.80
Age	−0.00086	0.00038	1	0.022	85	
Dominance	1.37	0.20	1	<0.001	85	
Social group size	−0.0042	0.085	1	0.96	85	
Natural sentinel data: is the dominance effect among males attributable to body condition?^a^						−1.65
Body condition	<0.00001	<0.00001	7	0.14	21	
Dominance	<0.00001	<0.00001	7	0.053	21	
Experiment 1: does an intrasexual challenge impact dominant male sentinel behavior?^a^						6.04
Observation session stage × playback treatment	Figure 2		27	<0.001	40	

The estimate and SE values are reported at the point where the fixed term was either removed from the model (with *P* > 0.05) or retained (with *P* < 0.05) under stepwise model simplification. Constant values reported from the minimum model (with only significant terms retained). df, degrees of freedom; SE, standard error.

^a^Values reported from a full glmmPQL model (see Methods for details).

#### Natural sentinel behavior: do dominant males invest differentially in sentinel behavior?

First, we investigated whether dominant males invest more in sentinel behavior than other dominance/sex classes, by modeling the factors that affect individual sentinel effort (measured in all analyses as the proportion of observation time that the focal individual spent sentineling, logit transformed) using a generalized linear mixed effects model with a normal error distribution. The fixed effects of primary interest were sex and dominance status, and to specifically test for differential sentinel effort by dominant males, we also fitted the interaction between these 2 terms. We also fitted social group size (number of individuals present in the social group during the observation session) in the initial maximal model to control for its potential influence on individual sentinel effort. Individual identity, observation session identity and observation session length (30 or 60min; see above) were fitted as random factors. This analysis included 200 measurements of 89 individuals from 25 social groups. Second, to investigate whether the higher sentinel effort of dominant than subordinate males detected in this first analysis could be attributed instead to an effect of age (as dominant males tend to be older; Harrison et al. 2013a), a generalized linear mixed effects model was performed using the subset of available data for males of known age (again with a normal error distribution and individual identity, observation session identity and observation session length fitted as random factors) with age, dominant status, and group size as fixed factors. This analysis included 85 measurements of 36 males of known hatch date from 21 social groups. Third, to investigate whether the higher sentinel effort of dominant than subordinate males could be attributable instead to an effect of body condition (using a separate sentineling data set with matched body condition measures; see description above), a generalized linear mixed model (GLMM) with a penalized quasi-likelihood estimator (glmmPQL) and a quasi-binomial error distribution was implemented in the package MASS in R (Venables and Ripley 2002), with observation session identity fitted as a random factor (individual identity was not fitted as there was only 1 measure per individual). The glmmPQL approach using quasi-binomial error distribution was favored to account for overdispersion.

#### Experiment 1: does an intrasexual challenge impact dominant male sentinel behavior?

To investigate the effect of the treatment (simulated intruder vs. control) on the sentinel effort of the resident dominant male, a GLMM with a penalized quasi-likelihood estimator (glmmPQL) and a quasi-binomial error distribution was again implemented in the package MASS in R (Venables and Ripley 2002). Observation session stage (pre- or post-playback) and playback treatment (male solo song or control track) were fitted as fixed effects along with the interaction between them, with resident pair identity fitted as a random effect. We used Spearman rank correlation to test for a relationship between the duet response of the resident pair (their total duration of duet song production during the 3-min playback, in seconds) to foreign male solo song and the post-playback sentinel effort of the resident dominant male for each group. Duet song was measured and subsequently analyzed, as this was the only song type produced during the experiment; the focal dominant male did not produce any solo song.

#### Experiment 2: does sentineling facilitate responses to an intrasexual challenge?

To investigate whether the resident pair’s latency to 1) first produce a duet and 2) first approach the playback tree differed between the 2 treatment contexts (resident male in a sentinel position vs. foraging), Wilcoxon paired tests were used.

## RESULTS

### Natural sentinel behavior: do dominant males invest differentially in sentinel behavior?

**Figure 1:**
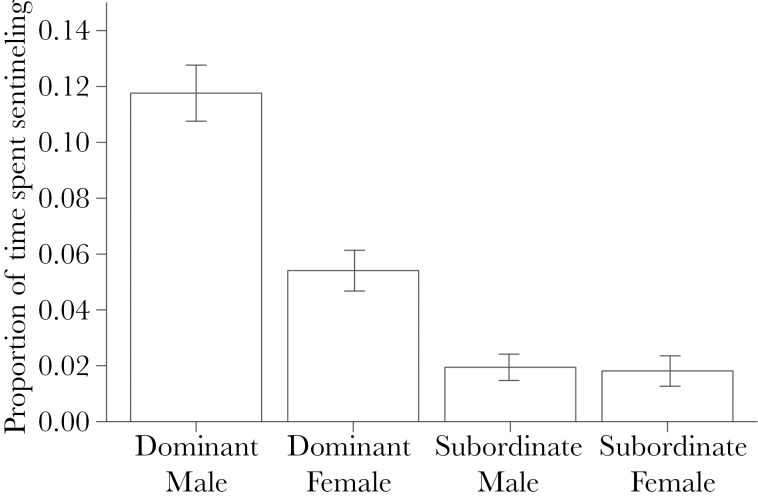
The contributions to sentineling of each dominance and sex class. Bars present mean ± standard error. The data were derived from 53 observation sessions on 25 groups, yielding a sample of 25 dominant males, 25 dominant females, 28 subordinate males, and 11 subordinate females.

During nonbreeding periods (times during the breeding season when groups lacked eggs and nestlings), foraging white-browed sparrow weaver groups had a sentinel in place a mean (± SD) of 21.0% (± 7.80%; *n* = 53 observation sessions of 25 groups) of the time, and sentinel bouts lasted a mean (± SD) of 179.44 (± 59.98) s (*n* = 200 bouts from 53 observation sessions). The sentinel bouts of group members were never observed to overlap, individuals were never observed being subjected to aggression while acting as a sentinel (*n* = 47 observation sessions of 20 groups had subordinates present, with 17 of these groups containing a subordinate male), and we had no reason to suspect that 1 individual had ever interfered with the sentinel efforts of another. The proportion of time that individuals contributed to sentinel activity was determined by a significant interaction between their dominance status and sex (GLMM: $\chi_{1}^{2}$ = 4.67, *P* = 0.031; Figure 1), and unrelated to group size ($\chi_{1}^{2}$ = 0.032, *P* = 0.86). Dominant males displayed significantly more sentinel effort than all other classes of individual, with dominant individuals exhibiting higher levels of sentinel behavior than subordinates (see Figure 1).

Two further analyses indicate that the elevated contributions of dominant males cannot be readily attributed to variation among males in age or body condition. Using the subset of data for males of known age (*n* = 85 measurements of 36 males of known hatch date from 21 social groups), a model of male sentinel effort confirmed that dominant males spent a significantly higher proportion of their time sentineling than subordinate males (GLMM: $\chi_{1}^{2}$ = 7.45, *P* = 0.006) even while controlling for a significant positive effect of male age on sentineling (GLMM: $\chi_{1}^{2}$ = 4.26, *P* = 0.039). Sentinel activity was unrelated to social group size ($\chi_{1}^{2}$ = 0.0026, *P* = 0.96). The mean (± SD) proportion of time spent sentineling for dominant males was 0.13±0.077, and for subordinate males, was 0.017±0.035. Utilizing data from incubation periods, when the body masses of dominant and subordinate males were assessed, there was no significant effect of body condition (as assessed using the SMI) on the sentinel contributions of males (glmmPQL: *t*_7_ = −1.66, *P* = 0.14) and, even with SMI retained in the model, the effect of dominance status approached significance despite the comparatively small sample size (*t*_7_ = −2.32, *P* = 0.053; *n* = 11 dominant males and 10 subordinate males of known SMI from 12 groups).

### Experiment 1: does an intrasexual challenge impact dominant male sentinel behavior?

**Figure 2:**
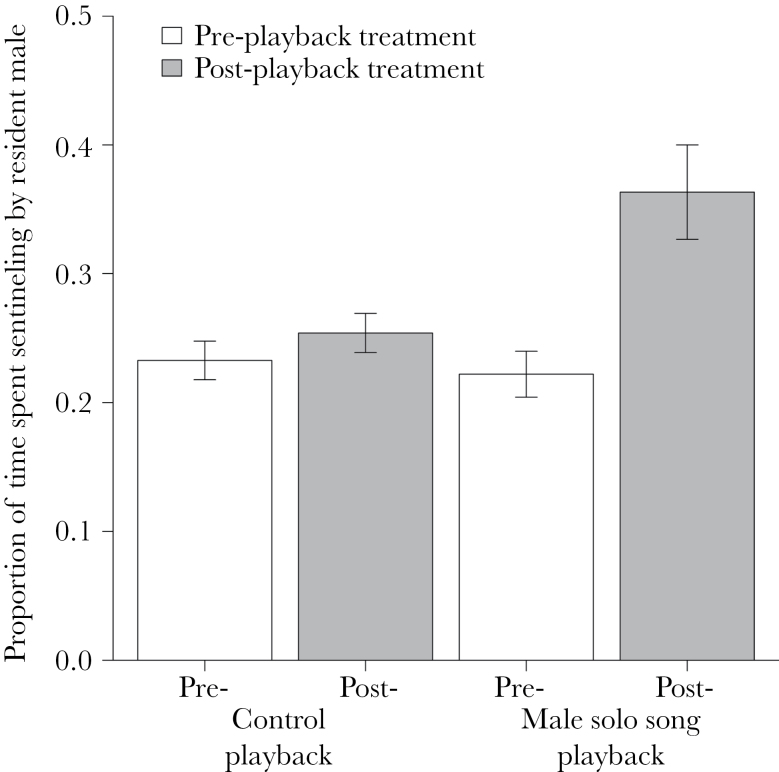
The sentinel activity of dominant males pre- versus post-playback of either the control or male solo song playback treatments (*n* = 10 groups). The bars present mean ± standard error. Unfilled bar, pre-playback; filled bar, post-playback.

**Figure 3:**
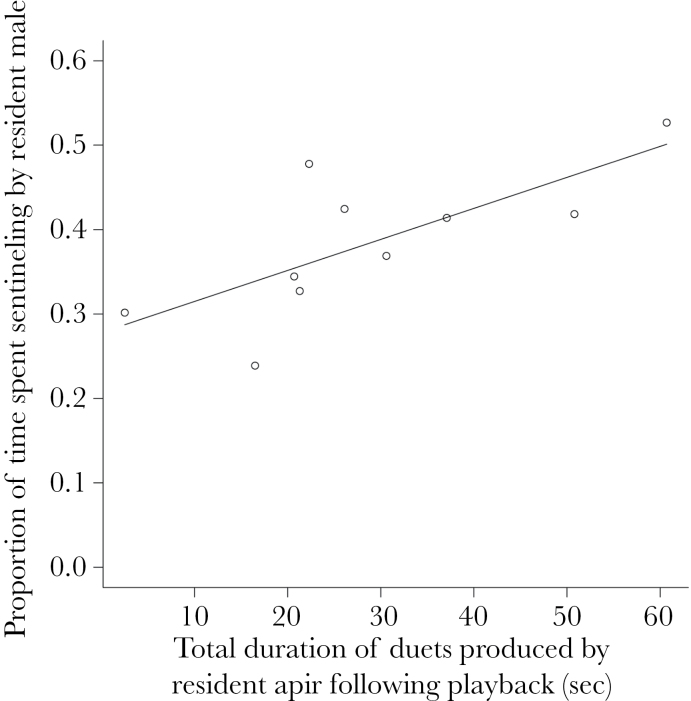
The total duet output of the resident pair in response to the playback of male solo song (*n* = 10 groups) positively predicted the subsequent sentinel effort of the resident male once the pair had returned to the foraging context.

The foreign male solo song playback elicited a robust duet response from the resident pair; they produced duets for a significantly longer total duration in response to the foreign male solo song playback than the control playback (interquartile range [IQR]: male solo song playback = 20.85–35.48s; control = 0.00–3.63s; Wilcoxon paired test: *V* = 55, *P* = 0.002, *n* = 10). The male solo song playback was then followed by a marked increase in sentinel behavior by the dominant male once the pair had returned to foraging, whereas the control playback was not, as indicated by a significant interaction between playback treatment (male solo song or control) and observation session stage (pre- or post-playback; glmmPQL: *t*_27_ = −6.52, *P* < 0.001; Figure 2). Across groups, the vocal response of the resident pair to the foreign male solo song playback (the total length of the duet song produced during the 3min playback) significantly positively predicted the dominant male’s subsequent sentineling effort (Spearman rank correlation: *r*_s_ = 0.78, *N* = 10, *P* = 0.012; Figure 3; trendline is for demonstration only). The dominant females did not contribute to sentineling in either treatment, before or after the playback (the playback was conducted during the incubation period, when dominant females rarely sentinel even when away from the nest). None of the dominant females engaged in incubation during the observation periods.

### Experiment 2: does sentineling facilitate responses to an intrasexual challenge?

**Figure 4:**
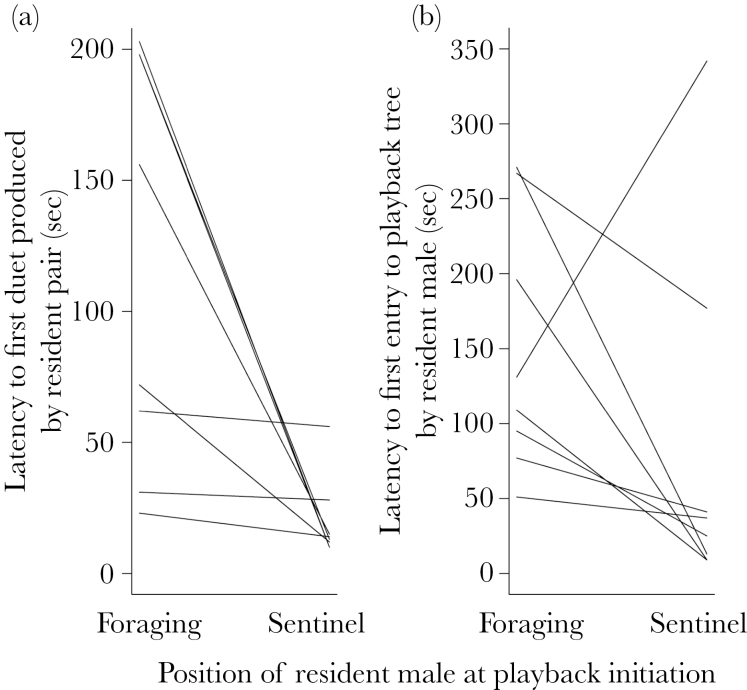
Latency to (a) first duet of resident pair and (b) first entry of dominant male to playback tree for dominant males exposed to male solo song in 2 contexts (sentineling vs. foraging; *n* = 8 groups).

When the 3-min foreign male solo song playback was initiated with the dominant male in a sentinel position, the resident pair produced their first duet a median of 13.50 (IQR: 11.50–18.25) s after playback initiation. When the playback was initiated while these same dominant males were foraging on the ground, the dominant males first moved to an elevated position on a nearby bush or tree 27.00 (IQR: 20.25–29.75) s after playback initiation, and the first duet was only produced a *further* 53.00 (IQR: 32.25–135.80) s after this movement. The resident pairs’ first duet was therefore produced with significantly shorter latencies from playback initiation if the dominant male was on sentinel at playback initiation rather than foraging (Wilcoxon paired test: *n* = 8 males, *V* = 36, *P* = 0.008; Figure 4a). The latency for the dominant male to enter the tree that contained the playback speaker was not significantly different between the 2 contexts (Wilcoxon paired test: *V* = 7, *P* = 0.15; Figure 4b), though in all cases but one the latency to approach the speaker was lower when the playback occurred while the male was sentineling. In all 8 groups and in both contexts, the dominant male entered the playback tree before the dominant female.

## DISCUSSION

We used a combination of natural observations and field experiments to investigate whether sentinel behavior may play a role in male intrasexual competition over mates and/or territory. Our findings reveal first that dominant male white-browed sparrow weavers (who face extra-group threats to both their paternity and social dominance; Harrison et al. 2013a, 2013b) display substantially more sentinel effort than other group members and that their differential contributions cannot be readily attributed solely to variation in age or body condition (SMI). Second, the playback of foreign male solo song (which would otherwise be indicative of the presence of an extra-group male) elicited a robust vocal response from the resident pair coupled with a movement response led by the dominant male, and this was followed by a marked increase in sentinel effort by the dominant male once the pair had returned to foraging, none of which were observed in paired control playbacks. Indeed, the magnitude of the vocal response to the foreign male solo song playback predicted the magnitude of the resident dominant’s subsequent sentinel effort. Finally, the resident pair also mounted significantly swifter duet responses to the foreign male solo song playback if the dominant male was acting as a sentinel at the time of playback initiation rather than foraging, suggesting that sentinel behavior may facilitate the rapid initiation of counter-intruder responses. Although research to date has primarily focused on an antipredator function for sentinel activity, our combined results suggest that sentinel behavior may also play a role in intrasexual competition and that sexually selected direct benefits may therefore have acted in concert with other mechanisms to shape contributions to sentinel behavior.

Consistent with the patterns of sentineling observed in Arabian babblers (Wright, Maklakovi, et al. 2001), dominant male white-browed sparrow weavers display higher levels of sentinel activity than any other class of individual. Several studies have now reported that heavier individuals contribute more to sentinel behavior (e.g., Arabian babblers: Wright, Maklakovi, et al. 2001; meerkat: Clutton-Brock et al. 2002; pied babblers: Bell et al. 2010) and have confirmed the state dependence of sentineling contributions with feeding experiments (meerkat: Clutton-Brock 1999; Arabian babbler: Wright, Maklakovi, et al. 2001; Florida scrub jay: Bednekoff and Woolfenden 2003). The elevated sentinel contributions of dominant male white-browed sparrow weavers cannot be readily attributed to variation in body condition, however, as 2 recent studies have examined dominance-related differences in body condition in this species, and, although both report evidence of such differences among females, no dominance-related differences in body condition were found among males in either the first or second halves of the breeding season in either study (Harrison et al. 2013b; Cram et al. 2015). Accordingly, when variation in body condition was statistically controlled in our model of male sentinel effort, dominance status approached significance (*P* = 0.053) despite the greatly reduced sample size. It is difficult to rule out any role for variation in nutritional state, however, as it remains possible that rank-related differences in hunger levels (conceivably independent of body reserves) could be contributing to the patterns observed. Several studies of sentinel behavior have also found that older individuals contribute more (e.g., Zahavi 1990; Hailman et al. 1994; Wright et al. 2001b; Wright, Maklakovi, et al. 2001), but the elevated efforts of dominant male white-browed sparrow weavers cannot be attributed simply to age-related variation, as dominant males showed significantly higher sentinel effort than subordinates while statistically controlling for effects of age. The differential efforts of dominant males also cannot be readily attributed to individuals interfering with the sentinel efforts of others (as envisaged by Zahavi A and Zahavi A 1997, but see Wright 1999), as we observed no evidence of aggression between sentinels or of 1 individual interrupting the sentinel bouts of another. The markedly elevated contributions of dominant males would not be predicted if sentineling instead played a role in the generalized defense of a resource territory, as dominant males and females might both be predicted to contribute at high rates in this scenario. Indeed, if sentineling did play a role in resource defense, the dominant female might be predicted to sentinel at the highest rate, as her reproductive success is likely to be more tightly linked to local resource availability than that of the dominant male (as dominant males can secure extra-group paternity). The most plausible explanation for the differential sentinel efforts of dominant males would therefore appear to be that they stand to gain differential direct benefits from sentineling as it may facilitate the detection and/or repulsion of same-sex competitors, in particular extra-group males (who intrude on territories during the day and are the principal threats to a male’s paternity and social dominance; Harrison et al. 2013a, 2013b; Harrison et al. 2014). Indeed, it is conceivable that the sentinel position also facilitates the monitoring of the resident dominant female’s movements, further facilitating mate-guarding.

The behavioral response to the playback of an unfamiliar male solo song is also consistent with the hypothesis that sentinel behavior plays a role in intrasexual competition. Immediately following the male solo song playback (and not following the control playback), there was a clear duet and movement response by the resident pair, the latter being led by the dominant male. Once the resident pair had returned to foraging, the dominant male substantially increased his investment in sentinel activity relative to the period prior to the playback, a change that was only evident following the male solo song playback and not the control playback. During the 52 sentinel bouts that the 10 focal dominant males conducted in the foraging period following the male solo song playback, the focal males never engaged in duet production with their mate and just once produced solo song. It seems unlikely therefore that sentineling in this context simply serves as a position from which to broadcast song (as suggested by Wright et al. 2001a, 2001b; Wright, Maklakovi, et al. 2001). Instead, our findings are consistent with the hypothesis that sentineling provides a vantage point that may facilitate the detection and monitoring of same-sex intruders. As the experiment was conducted during the incubation period, the resident male’s response could have been motivated more by the defense of his dominant position than by a risk of lost paternity. It is also worth noting that the response to the solo song playback cannot be attributed specifically to our use of solo song per se, as it could instead reflect a generalized response to the detection of an unexpected conspecific (via cues within the song; Townsend et al. 2012). The dominant male’s sentineling response to the male solo song playback cannot be attributed instead to compensatory responses (or indeed coordination responses) by the dominant male due to changes in the sentineling effort of other group members, as the only group member present was the dominant female (as the playbacks were conducted on resident pairs, to rule out the complications posed by helper responses), and the dominant female did not modify her sentinel effort in response to either playback (she contributed nothing before and after both treatments, which is not uncommon during the incubation period; Walker LA, Young AJ, unpublished data).

It is quite possible that the dominant male’s sentinel response to the foreign male solo song playback is a response in part to the dominant female’s own vocal response to the playback, as although the dominant male clearly led the movement response to the playback, the concomitant marked increase in duet production could have been led by either the dominant male or female (duet production is so synchronous that the leaders of duets cannot be readily identified in the field). Although duets are frequently interpreted as cooperative vocal responses that may function in the collective defense of territory (e.g., white-browed sparrow weaver: Ferguson 1988; Wingfield and Lewis 1993; Voigt et al. 2006), they may also reflect sexual conflict (Marshall-Ball et al. 2006; Tobias and Seddon 2009), in which 1 sex may advertise their presence to putative extrapair mates, eliciting an immediate response from their social partner that may serve in mate defense. Our finding that the duet response of the resident pair to the male solo song playback positively predicted the dominant male’s sentinel response is consistent with this view, with the dominant male potentially scaling his subsequent sentinel response according to the dominant female’s vocal response to the playback. Alternatively, this positive association could reflect a shared anti-intruder function for both the duet and sentinel responses, with the expression of both being modulated according to individual variation in the male’s expected payoffs from repelling intruders or simply according to variation in his nutritional state.

Consistent with the hypothesis that the sentinel position confers an advantage in detecting and responding to intruders, our second experiment revealed that the latency from the initiation of foreign male solo song playback to the production of the first duet by the resident pair (a song type that functions in territorial defense; Wingfield and Lewis 1993; Voigt et al. 2006) was significantly lower if the playback was initiated while the dominant male was sentineling rather than foraging on the ground. The delay in the duet response when dominant males were foraging rather than sentineling at playback initiation could be due in part to 1 or more of the following mechanisms: 1) Males may simply tend to be in a poorer nutritional state when foraging than when sentineling (given the likely state dependence of sentineling; Wright et al. 2001a, 2001b; Wright, Maklakovi, et al. 2001; Bednekoff and Woolfenden 2003) and so may differentially value continuing to forage relative to mounting a counter-intruder response; 2) mounting an immediate counter-intruder response might entail an additional lost-opportunity cost for foraging males if they have to interrupt an active foraging attempt; and 3) males that are foraging in cover may simply take longer to detect the playback than a sentineling male on an elevated perch. Although each of these mechanisms could explain why foraging dominant males take time following playback initiation to cease foraging and rise from the ground, none of them can readily explain why the delay from this point to the first duet produced was still significantly longer than the entire delay from playback initiation to first duet production for males that were sentineling at playback initiation (see Results for details). This discrepancy suggests that foraging males, even after they have ceased foraging, may first take time to assess their environment and/or the location of the intruder before mounting a vocal response, whereas sentineling males may require little time for assessment as they have already been continuously monitoring their environment. Sentineling may therefore yield benefits in intrasexual competition by facilitating both the efficient detection of intruders (whether visually or acoustically) and the subsequent mounting of swift responses.

Although our results are consistent with sentinel behavior simply facilitating the detection and repulsion of same-sex intruders, it is conceivable that sentinel behavior also serves as a signal in this context (Zahavi 1995; Zahavi A and Zahavi A 1997; Wright 1999). Sentinel behavior could, for example, highlight the presence of a resident dominant to passing would-be challengers, which could itself be sufficient to prevent intrusions in many cases. But where sentineling entails costs (e.g., via lost foraging time and/or exposure to risk, e.g., Ridley et al. 2013), it has also been hypothesized to serve as an honest signal of quality (e.g., Zahavi 1995; Zahavi A and Zahavi A 1997; Wright 1999). The differential sentinel effort of dominant males and their sentinel responses to same-sex intruders could therefore also be interpreted in this light; dominant males could conceivably be signaling their quality in both cases, to same-sex competitors and/or their social mate, both of which could act in concert with improved intruder detection to further promote success in intrasexual competition. Although it has been suggested that a signaling role for sentineling might also ultimately lead to within-group competition over sentinel contributions (Zahavi A and Zahavi A 1997, but see Wright 1999), we found no evidence to suggest that this was the case, consistent with the arguments of Wright (1999). Whether sentineling does indeed serve as an honest signal of quality in this or other species remains to be investigated.

Previous research has highlighted the role that sentinel behavior may generally play in mitigating predation risk in social groups, conceivably both for the actor and their fellow group members (e.g., Wright et al. 2001a; Ridley et al. 2010; Santema and Clutton-Brock 2013), leading to debate over the relative roles that direct and kin-selected indirect benefits have played in the evolution of this potentially cooperative behavior. Together, our findings support the hypothesis that sentineling may also play a role in intrasexual competition, potentially facilitating the effective defense of both paternity and dominance status against extra-group challengers. Indeed, observations that males in a number of other species also invest differentially in sentinel behavior (e.g., vervet monkeys: Baldellou and Peter Henzi 1992; Florida scrub jays: Hailman et al. 1994; Arabian babbler: Wright, Maklakovi, et al. 2001; meerkats: Clutton-Brock et al. 2002), highlights the possibility of a more widespread role for sentinel behavior in male–male competition. Although our investigations have focused on males, females too may stand to benefit directly from investment in sentineling if it facilitates the detection and repulsion of same-sex competitors. Although dominant females may rarely lose parentage to transient same-sex intruders (as dominant males frequently do), competition among females for the dominant position per se is frequently intense in cooperatively breeding species (Clutton-Brock et al. 2006; Young and Bennett 2013). Although sentinel behavior is frequently assumed to reflect an example of cooperation (specifically, a behavior that provides a benefit to another individual (recipient) and that is selected for because of its beneficial effect on the recipient; West et al. 2007), the criterion that selection for sentineling arises because of its beneficial effect on recipients has to our knowledge yet to be conclusively demonstrated (Clutton-Brock 1999; Ridley et al. 2013; Santema and Clutton-Brock 2013). Indeed, our findings only add to the challenge of demonstrating this, as while evidence that sentinels are exposed to greater predation risk than non-sentinels might suggest that sentineling entails a direct fitness *cost* (Ridley et al. 2013), the possibility that sentineling also yields direct fitness *benefits* through mechanisms unrelated to predation (such as benefits in intrasexual competition) leaves it more difficult to reach this conclusion. To the extent that sentineling does indeed reflect cooperative behavior, our findings lend new strength to the view that direct fitness benefits can shape the expression of cooperative behavior and highlight the wider potential for sexual selection to act in concert with more-commonly invoked mechanisms in shaping patterns of cooperation.

## FUNDING

L.A.W. was supported by a NERC PhD studentship (NE/J500185/1). J.E.Y. was supported by a BBSRC postdoctoral position (BB/H022716/1) and a University of Pretoria Postdoctoral Fellowship. A.J.Y. was supported by a BBSRC David Phillips Fellowship (BB/H022716/1).
